# School-Based Integrated Care Within Sydney Local Health District: A Qualitative Study About Partnerships Between the Education and Health Sectors

**DOI:** 10.5334/ijic.7743

**Published:** 2024-05-02

**Authors:** Santuri Rungan, Jennifer Smith-Merry, Huei Ming Liu, Alison Drinkwater, John Eastwood

**Affiliations:** 1 Croydon Health Centre, 24 Liverpool Road, Croydon, NSW, AU; 2 Centre for Disability Research and Policy (CDRP), Susan Wakil Health Building (D18), Camperdown Campus, The University of Sydney, NSW, 2006, AU; 3 Menzies Centre for Health Policy, University of Sydney, AU; 4 The George Institute for Global Health, University of New South Wales, AU; 5 School of Population Health, University of New South Wales, Kensington, NSW 2050, AU; 6 Sydney Local Health District, Level 11, King George V Building, Missenden Road, Camperdown NSW 2050, AU

**Keywords:** School-based integrated care, Integrated People-Centred Health Service (IPCHS) framework, implementation of integrated care

## Abstract

**Introduction:**

The unmet physical and mental health needs of school-aged children (5–18 years) in New South Wales (NSW), stemming from poor access and engagement with healthcare, can be addressed by school-based integrated care (SBIC) models.

This research aims to understand why and how partnerships between the health and education sector, in SBIC models, are important in providing care for children, and to identify the facilitating factors and barriers for implementation.

**Methods:**

A qualitative study was conducted using semi-structured interviews and thematic analysis. The principles of the ‘Integrated People-Centred Health Service (IPCHS)’ framework and Looman et al’s (2021) implementation strategies for integrated care were considered.

**Results:**

Themes within IPCHS framework: Strategy 1: Engaging and empowering people and communities – community-driven models, improved access to healthcare, positive outcomes for children and families, ‘connection’, and service provision for marginalised populations; Strategy 2: Strengthening governance and accountability – system integration and developing evidence base; Strategy 3: Reorienting the model of care – shifting healthcare to schools reduces inequity and provides culturally safe practice; Strategy 4: Coordinating services within and across sectors – integrating care and stable workforce; Strategy 5: Creating an enabling environment: leadership, stakeholder commitment, and adequate resourcing.

**Discussion:**

Potential strategies for implementing SBIC models across NSW include community consultation and co-design; building multidisciplinary teams with new competencies and roles e.g. linkers and coordinators; collaborative and shared leadership; and alignment of operational systems while maintaining a balance between structure and flexibility.

**Conclusion:**

SBIC models require high-level collaboration across sectors and with communities to provide a shift towards child and family centred care that improves engagement, access and outcomes in health delivery.

## Introduction

School-aged children (5–18 years) frequently have unmet physical and mental health needs due to poor access and engagement with health services [1,2,3]. This issue disproportionately affects Aboriginal children and children living in rural New South Wales (NSW) [4,5,6]. School-based healthcare (SBHC) has re-emerged in Australia as a mechanism of integrating education, health and wellbeing services [7,8]. Internationally, similar models show improved health outcomes, education outcomes, access to healthcare, high acceptability, and favourable cost-benefit ratios [9,10,11].

In Sydney Local Health District (SLHD), an integrated model of care has been established with schools and the community [12]. Integrated care can be described as the bringing together of the fragmented parts of a health system to optimise care [13,14]. The SLHD model provides comprehensive and holistic health and wellbeing assessments for children delivered at local schools by a multidisciplinary team with representatives from the health, education and social work sector [12]. The value of integrating care by collaboration across sectors has led to the model being called ‘school-based integrated care (SBIC)’. The pilot for the initiative, Ngaramadhi Space (NS), was established at a school for students experiencing problematic behaviour (Yudi Gunyi School (YGS)) [12]. A quantitative evaluation of NS showed improved access to healthcare for students with high attendance rates and significant improvements in teacher reported behavioural scores [15].

The NS model of care has been replicated in four schools within SLHD under the name ‘Kalgal Burnbona’, meaning ‘to surround family’ in the Dharawal language of the local Aboriginal community. Other SBIC models have independently emerged across NSW [7]. The ground swell of interest in implementing SBIC has led to the formation of a community of practice (COP).

**Figure 1:**
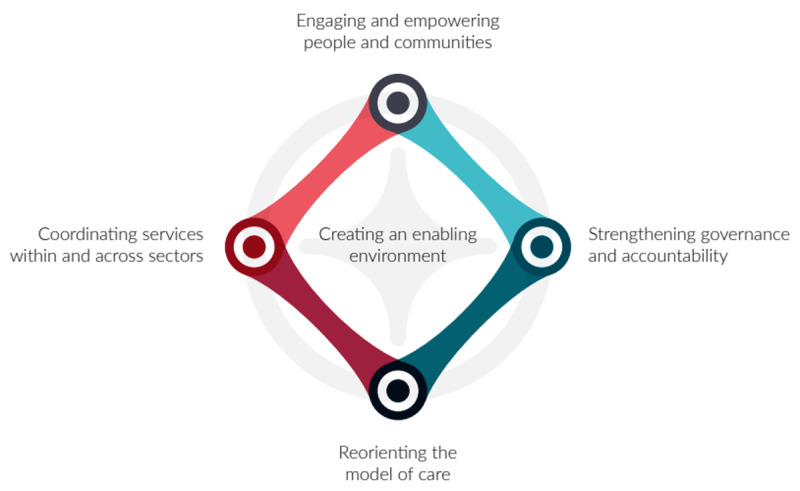
The five interdependent strategies of the WHO Framework on integrated people-centred health services (IPCHS) [14].

**Figure 2:**
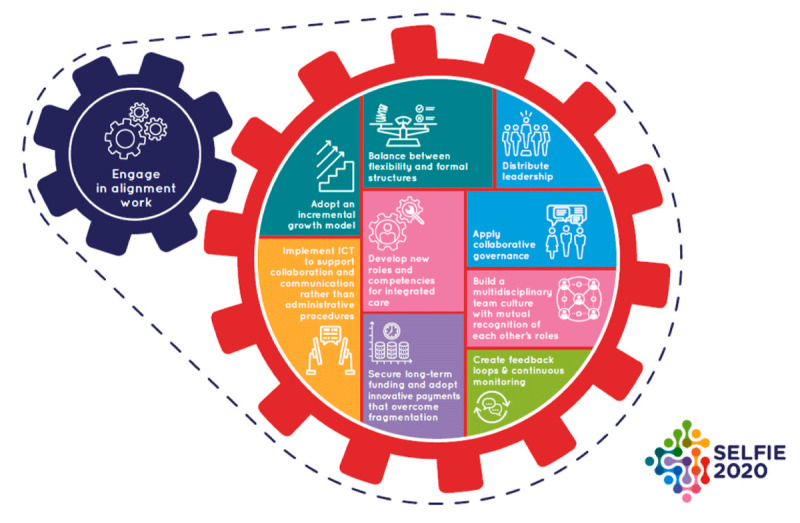
The 10 implementation mechanisms for integrated care for multi-morbidity [16].

The aim of this qualitative research study was to understand why and how partnerships between the health and education sector, in SBIC models of care such as NS, were important in providing care for children, and to identify the facilitating factors and barriers to this process. To assist with this understanding, the principles of the ‘Integrated People-Centred Health Service (IPCHS)’ framework (Figure 1) and Looman et al’s (2021) underlying implementation strategies for integrated care (Figure 2) were considered [16]. The IPCHS framework proposes five strategies to transform health service delivery so that it is more responsive to people’s needs [14]. Looman proposed ten implementation strategies for scaling up integrated care initiatives (Figure 2) [16]. The IPCHS framework was used to contextualise the main themes from the data analysis while Looman’s implementation strategies were used to derive mechanisms for broader scaling up of the SBIC model [14].

## Research Methods

### Study Design and Methodology

**Figure 3:**
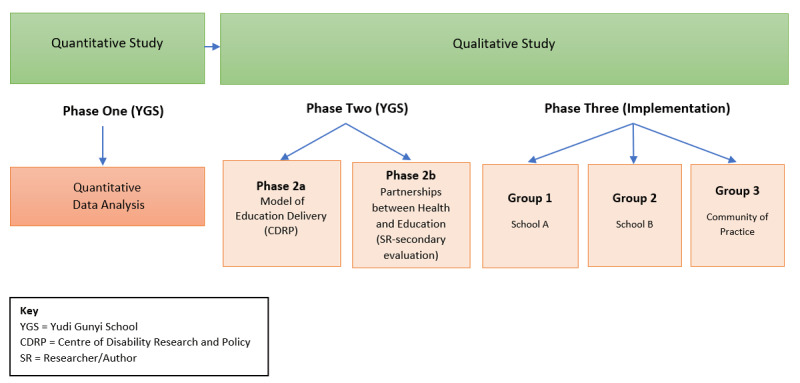
Diagram showing the design of the research study.

A critical realist-informed three-phase, sequential mixed method study was designed (Figure 3). Critical realism is a philosophical system where ‘realism’ describes a natural world that exists outside of our interpretations of it, and ‘critical’ refers to the study of science through the interactions of human language and social powers over time [17,18]. Critical realism is relevant to this research because SBIC has emerged as a potential solution for inequitable health, education and social outcomes observed within local communities. This study aims to understand the ‘nature’ or mechanisms behind SBIC models, particularly the facilitating factors and barriers to this process.

Phase 1 was a quantitative study of NS that has been described previously [15]. The qualitative research was conducted in two phases using the COREQ checklist [19]. Phase 2 involved YGS and was undertaken in two parts with a focus on the model of education delivery, and partnerships between the health and education sector. Phase 3 studied how the SBIC model had been replicated at other sites and focused on understanding the barriers and facilitators to successful implementation.

### Setting and Participants

Phase 2 was conducted at YGS by two researchers from the Centre of Disability Research and Policy (CDRP), including an Aboriginal researcher (August–September 2022). Participants included Aboriginal community members, education leaders, teachers, NS team members, students and parents. Phase 3 was conducted by SR with two of the four SLHD sites that had implemented the NS model, and the COP (November 2022–February 2023). Participants included paediatricians, school counsellors, school principals, social workers and other stakeholders.

### Recruitment, Interviews and Data Management

Phase 2 participants were contacted by the YGS social worker. Phase 3 participants were contacted by a research assistant with the first two schools to agree to participate included. After written consent was received, semi-structured interviews were conducted in person or via videoconferencing using audio recording and an interview guide (Appendix A and B). Support people were permitted, and participants could review and edit their interview transcript prior to analysis. All participant information sheets clearly stated confidentiality and voluntary participation. Table 1 summarises the anticipated and actual number of participants.

**Table 1:** Table showing the anticipated and actual number of study participants.


SITE	PARTICIPANT GROUP (ABBREVIATION)	ACTUAL PARTICIPANTS/ANTICIPATED PARTICIPANTS

Ngaramadhi Space (NS)	School Principal (SP)	1/1

	School Executive (SE)	2/2

	School Teacher (ST)	6/7

	Social Worker (SW)	1/1

	Occupational Therapist (OT)	1/1

	Speech Pathologist (SP)	1/1

	Paediatrician (PD)	1/1

	Aboriginal Community Member (ACM)	2/3

	School Student (SS)	3/5

	Parents/Caregiver (PC)	0/4

School A	School Counsellor (SC)	2/2

	Social Worker (SW)	2/2

School B	School Principal (SP)	1/1

	School Counsellor (SC)	1/1

	Paediatrician (PD)	1/1

Community of Practice (COP)	Director of Community Paediatrics (DCP)	1/2

	Rural General Practitioner (RGP)	1/1

	Director of Non-Governmental Organisation (DNG)	1/1

	Researcher (RES)	1/2

	**Total**	**29/39**


### Data Analysis

De-identified data was analysed using Braun and Clarke’s Thematic Analysis Framework where the methodological study of patterns within qualitative data to uncover meaning are described [20]. A reflective process was undertaken after each interview through note-taking or debriefing to identify emerging themes [21]. Data was manually ‘coded’ using Nvivo qualitative data analysis software [22]. SR and HML held in-depth discussions after the initial thematic analysis to triangulate and reach agreement on emerging findings. SR is a dual-trained general paediatrician and community paediatrician with a Master of Public Health and Tropical Medicine. HML is public health physician with a Master of Public Health and a PhD.

The ontological and epistemological views of both researchers were positioned in the middle with the importance of both objective and subjective information being recognised. Utilising a critical realist approach, abductive reasoning, meaning the application of logical inference to seek the simplest and most likely conclusions, and retroductive reasoning, meaning to use prior knowledge and available evidence to explain an observation, was used to then identify themes within the WHO’s five strategies and emerging strategies around implementation [23]. The next stage of analysis involved further triangulation, abductive and retroductive reasoning to align these themes with Looman’s implementation strategies. Care and a reflexive approach were taken to avoid over-interpretation of quotes and themes that would lead to a bias where data was made to ‘fit’ into the frameworks described [21].

### Ethical Considerations

To ensure the safety and comfort of the students involved, support persons were permitted during interviews, and the researchers were experienced in working with students.

As a clinician researcher, SR mitigated the risk of coercion by having a research assistant contact potential participants. Deidentified data was provided to SR for Phase 2 to reduce the risk of identifying participants. Furthermore, school names have been de-identified with the exception of YGS/NS because this school has appeared in earlier publications. For NS, the persons in each role have changed many times, which reduced the risk of identifying individuals. Additionally, all participants could review and edit their transcripts. Ethics approvals were received from SLHD Human Research Ethics Committee (SLHD HREC), Aboriginal Health and Medical Research Council (AH&MRC) and State Education Research Applications Process (SERAP).

## Results

### Participants

De-identified data from all 18 participants from NS (Phase 1) was included in the analysis. In Phase 2, there were 4 and 3 participants from School A and School B respectively. There were 4 participants from the COP who discussed SBIC models in Southwestern Sydney Local Health District (SWSLHD), Illawarra Shoalhaven Local Health District (ISLHD) and Southern NSW Local Health District (SNSWLHD). The characteristics of the SBIC programs are described in Table 2.

**Table 2:** Characteristics of schools included in this study.


SCHOOL CHARACTERISTICS	SYDNEY LOCAL HEALTH DISTRICT			SOUTHWESTERN SYDNEY LOCAL HEALTH DISTRICT	ILLAWARRA SHOALHAVEN LOCAL HEALTH DISTRICT	SOUTHERN NSW LOCAL HEALTH DISTRICT

	SCHOOL A	SCHOOL B	YUDI GUNYI SCHOOL			

**Type of School**	Government	Government SSP*	Government SSP*	Government	Government	Government

Year Group (K-12)**	K-12	K-6	6–12	K-6	K-6	7–12

**Location**	Major Cities NSW	Major Cities NSW	Major Cities NSW	Major Cities NSW	Inner Regional NSW	Outer Regional NSW

**Number of students (n)**	1034	34	27	287	454	563

**Female (n %)**	448 (43%)	6 (18%)	6 (22%)	138 (48%)	223 (49%)	253 (45%)

**Male (n %)**	586 (57%)	28 (82%)	21 (78%)	149 (52%)	231 (51%)	310 (55%)

**Indigenous students (%)**	17%	24%	52%	13%	51%	12%

CALD background***(%)	57%	43%	25%	75%	4%	5%

**ICSEA score** **and centile^**	1037 (64th)	934 (18th)	851 (4th)	880 (7^th^)	783 (2%)	946 (23^rd^)

**SEA Distribution^^**						

– **Bottom quartile (25%)**	19%	46%	63%	71%	80%	48%

– **Middle quartile (25%)**	19%	23%	24%	20%	14%	31%

– **Middle quartile (25%)**	28%	16%	9%	8%	5%	14%

– **Top quartile (25%)**	34%	16%	4%	1%	1%	6%

**IRSD^^^** **1 = most disadvantaged** **5 = least disadvantaged**	5	3	4	1	1	1


*SSP = School for Special Purposes. **K-12 = Kindergarten to Year 12. ***CALD = Culturally and linguistically diverse. ^ ICSEA = Index of Community Socio-Emotional Advantage. The Australian average is 1000 [24]. ^^ SEA = Socioeconomic Advantage. The Australian average is 25% for each quartile [24]. ^^^IRSD = Index of Relative Socio-Economic Disadvantage (IRSD) ranking for surrounding suburbs to school [25].

### Analysis

#### Strategy 1: Engaging and Empowering People and Communities

Engaging and empowering people and communities relates to providing the opportunity, skills and resources for people and communities to make effective decisions about their own health and their role in producing health-promoting environments. This strategy speaks to the ability to reach and co-design health services for marginalised populations [14]. The following themes relating to this strategy were derived from the data.

##### Theme 1: Community-Driven Models of Care

Participants described the community-driven development of SBIC models across NSW. Although the models were developed independently of each other, they all involved collaboration between the health, education, and social care sectors with input from the community. For example, the ISLHD model was driven by the community because of concerns that students were missing school to attend medical appointments, “*So the solution then was to have a physical hub within the school*” [RES]. In SWSLHD, a NGO developed a SBIC to foster community connections in an area experiencing high social disadvantage:

*“Belonging to community, belonging to school…trying to create that sense of connectivity in the community.*” [DNG]

SNSWLHD is a rural area where a GP-led SBIC was developed to improve access to paediatric care in the context of “*a very traumatised community with multi-generational trauma, people from the stolen generation… quite high rates of domestic violence, crime and social housing*” [RGP] as well as geographic isolation and being impacted by bush fires, flooding and the Covid pandemic.

##### Theme 2. Improved Access to Healthcare

Across all sites, participants described how SBIC programs addressed the challenges of engaging and navigating mainstream health pathways which included waiting lists, fee-for-service models, complex processes, and inflexible eligibility criteria. Positioning health services at schools helped remove these barriers with schools perceived as being familiar, convenient, and trusted. Families seen at SBIC sites were described as experiencing “*a sense of relief at finally being able to access care*.” [RES] This was particularly noticeable for priority populations who would have otherwise “*slipped through the gaps*” [SW1] or those who were reluctant to access healthcare in a mainstream setting:

*“An example would be a victim of the stolen generation. They’re not gonna want to go into a big, scary hospital to try and access care for their child, whereas they’re used to taking their kid to the school. You know it’s much less threatening.”* [RGP]

##### Theme 3. Positive Outcomes for Children and Families

Children benefited from accessing health services at school. This included receiving diagnostic and therapeutic support for behavioural, developmental and physical health concerns. For example, one student received treatment for a recurrent sexually transmitted infection, while the multidisciplinary approach prevented another child from being referred to a specialised behavioural school. The following quotation explains these types of benefits:

*“We found improvements in the [Strengths and Difficulties Questionnaires] of kids [that] were maintained…and in quality of life… The mental health of the parents and their self-efficacy also improved. We saw a protective effect in terms of behaviour[al] incidents in schools.*” [RES1]

##### Theme 4: Connection

A sense of connection was facilitated by SBIC. Health and education staff recognised the importance of building relationships with children to help with engagement, while acknowledging that relationships between the health and education sectors helped foster a better understanding of students. The importance of building connections is illustrated below:

*“I see part of my role as trying to understand the child’s story”* [PD2]

*“I say that to our staff, you’ve got to make connection to the kids – It’s just about having conversations and yarns… just those simple little things can change a kid’s life.”* [AC2]

*“Just to give them the best opportunities and also a bit of care, that care and kindness… It takes a long time to build that trust and … then you can actually start moving to those next steps.”* [ST3]

Connection included engaging the *“whole of family”* [DNG] by providing better support to students in the context of their family and social circumstances. One participant believed that schools were a place that students could belong to, describing how allowing students to name a school allowed for this:

*“You take the name; the kids pick the name. Yudi Gunyi means ‘place of learning’, they chose that. That gave them a sense of real responsibility … Before it was [called] Yudi Gunyi, …it was called Green Square Behaviour School. That’s like saying a prison straight up.”* [AC1]

##### Theme 5. Marginalised Population

Those seen at the various SBIC programs were described as underserved or marginalised because of the nature of their externalising behaviours and experiences of adverse childhood events. This made it difficult for them to engage with traditional health care services as exemplified in this quote:

*“They’re so complex and there’s so much going on in their lives that they shouldn’t be managing at that age or dealing with. Then they’ve got school on top of that…I like the fact that we’ve created this environment where these kids that just would fall through the cracks or whatever, they feel safe enough to come to a school setting.”* [ST2]

At times, the level of challenging behaviour experienced in the school seemed to have an impact on staff wellbeing:

*“Well [this] is definitely the most challenging school I’ve ever worked in…Here’s the hardest, the most violent, without a doubt, externalising violent behaviours.”* [ST3]

At times, the level of challenging behaviour experienced in the school setting led to medical staff feeling pressured to make immediate changes to a child’s medication regime in response to problematic behaviour. Similarly, social workers felt that in advocating for the needs of children, staff did not always recognise the boundaries of the social worker’s role. This created tensions around expectations from those involved.

#### Strategy 2: Strengthening Governance and Accountability

Strategy 2 of IPCHS framework discusses the importance of strengthening governance across all levels of the health system. Good governance is described as transparent and inclusive with efficient use of resources reinforced by a system for accountability. For SBIC, governance and accountability involved both the health and education sector and the complexities of this partnership are described under the following themes [14].

##### Theme 1. Integration of Systems

The health and education sectors were described as complex entities which made integration of systems for the purposes of governance challenging. Each sector had their own values and perceptions while operating within different hierarchical processes and systems. This led to lengthy processes in receiving approval to share information and to sign off documents e.g. Memorandum of Understanding (MOU) and contracts. Participants spoke of a ‘silo’ effect where each sector worked independently of each other with a paucity of understanding about what other sectors did.

##### Theme 2. Developing a Robust Evidence Base

For SBIC models to flourish and to build transparency and accountability, participants agreed that a robust evidence base was needed. To do so, clinically relevant indicators were required, and evaluation processes had to be embedded in the model. The challenges of evaluating SBIC was elaborated on and included agreement on outcome measurements and stable resourcing. Participants described challenges in defining ‘success’ because positive gains would not necessarily translate into better academic achievement:

*“Because it’s not students reaching these major academic goals or what have you. Success would be –it’s very individualised. Would be just them having confidence in themselves.”*[ST3]

*“It’s important they can come here and have wins, and even if those wins are tiny little incremental wins- it’s taken a year for a girl to take off her double hoody… So, they’re little, tiny wins that are huge.”* [ST2]

Participants spoke about the difficulties associated with obtaining research consent in a clinical setting:

*“It feels like in clinic that you’re on a tightrope of sitting okay with the family … but you can read the body language if it’s becoming uncomfortable. And that’s sort of where I feel like consent around research gets you to.”* [PD1]

#### Strategy 3: Reorienting the Model of Care

Reorienting the model of care refers to a shift away from hospital-based healthcare to community-based healthcare by designing comprehensive and innovative models of care. In this section a shift towards basing healthcare at schools is highlighted as is the importance of ‘one-stop shops’ to the Aboriginal community.

##### Theme 1. Shifting Healthcare to Schools

SBIC models were seen as a mechanism to deliver a comprehensive lifespan approach to health and wellbeing that *“pivots and bends”* [RGP] to the changing needs of individuals. Basing health services at schools allowed more readily available access to care, creating an avenue to reduce inequity, “*A service delivered where it needs to be delivered for vulnerable children and families”* [RGP].

Participants, particularly Aboriginal community members, highlighted the value of having “*one stop shops*” [AC2]. This meant having all the services required by families located in the one place. Co-location though was not enough, and it was important for those services to work together to develop a holistic understanding of the child and family. Participants spoke about the importance of building a collaborative multidisciplinary culture, “*as [the team has] evolved, yeah, rehashing those conversations and re-establishing those expectations of each other”* [SW1]. The concept of a core multidisciplinary team that worked together was discussed along with the possibility of one group working across several schools:

*“To scale it up…there needs to be a social worker, perhaps a mental health professional …and occupational therapist and speech [pathology] and the paediatrician…We would have one worker across multiple schools, and just doing those paediatric assessments with health and with education [and] we’ve got all of those insights as part of that assessment…”* [SW1]

##### Theme 2. Cultural Safety

The SBIC models were co-designed with local Aboriginal communities and described as improving access to healthcare for Aboriginal children. AC2 discussed how it could be difficult for Aboriginal families to engage with services:

*“If it’s speech or hearing, vision, whatever. We try to make sure that that happens, but that’s not always easy… I’m always about connecting with the families, you know, those services are out there, but they need to make a connection with the families.*” [AC2]

Some culturally-safe practices included displaying Aboriginal artwork or signage and employing Aboriginal staff. Participants spoke about how important it was for Aboriginal families to see familiar faces, and for professionals to take the time to build trust, communicate, and help individuals feel comfortable with a service. The following quote describes these features:

*“[Aboriginal] people that live in the community, they are here for years, whichever medical centre they use, they go back because they feel comfortable and they can see your face. That they know there’s not gonna be made judgment upon them or anything”* [AC2]

#### Strategy 4: Coordinating Services Within and Across Sectors

In Strategy 4 the importance of coordination within and across services to meet the needs of people are highlighted. This requires integration of systems including referral pathways and linkages across sectors to optimise resources and align processes [14]. In this section themes relating to how care was integrated within the SBIC models is elaborated on including barriers to this process.

##### Theme 1: Integrating Care

In a SBIC setting, participants unanimously valued the range of expertise and skill sets provided by a multidisciplinary approach including accessing diagnoses, treatment, referral pathways and social support. SBIC teams worked collaboratively by a process of sharing information and joint assessments. This allowed for a holistic understanding of families, as exemplified in the following quote:

*“The Ngaramadhi Space is the health program that’s part of the school which has the school counsellor, …paediatrician, nurse, social workers, OT and speech and art therapy and it works in conjunction with the school to support the kids in all of their needs. So it’s really holistic so that [students] are able to flourish and ideally transition back to mainstream [schooling].”* [SE2]

Integrating care was seen as a more efficient way of working and allowed for timely transfer of information and a sense that “*we’re covering all bases to support the students to learn and engage most effectively…*” [ST3].

One participant discussed how the multidisciplinary approach helped support students while “*building parents’ and community members’ confidence in the education system”* [ST2]. Teachers felt that the clinical assessments provided them with *“a snapshot of what’s happened to this child. Understanding their triggers; understanding their point of view*” [ST2].

Participants described professional benefits from an integrated approach which included sharing of responsibilities and workload as well as knowledge exchange. For example, school counsellors valued having access to a paediatrician to discuss complex needs with and drew upon the paediatrician’s knowledge to use new tools. Paediatricians described collaboration with schools as a powerful tool for diagnostic and therapeutic purposes:

*“…the teachers see a lot of the features that [we] don’t hear about … And these kids are really complicated…And I [make observations] on the playground …And that’s an opportunity that we don’t normally get, which is really fantastic.”* [PD1]

Teachers and health staff described the pivotal roles played by school counsellors and social workers within SBIC models. School counsellors were able to bridge the gap between the health and education sector, in part because they understood the ‘language’ spoken by each sector but also because of their communication and relationship-building skills. The social worker and school counsellor were seen as playing essential roles in coordinating care as exemplified in this quote:

*“In schools with children who are this complex, there is a need to link in with health…And I think a school counsellor could be [in that role].”* [SP2]

Education staff were admired by the various sectors for their ability to build trust and facilitate engagement with families as well as to anticipate emerging issues, as shown in the following quotation:

*“[The] line of sight that the school has cannot be underestimated. They can anticipate months before [child protection services] that a child’s wellbeing is on the downhill.*” [DNG]

A key feature associated with integrating care was building on existing partnerships within the community to utilise resources more efficiently. For example, where available Wellbeing Health In-Reach Nurse (WHIN) coordinators and youth workers were integrated into SBIC models.

Challenges associated with cross-sector work were identified. Each sector had their own language and often worked independently of each other. The following quotes illustrate this finding:

*“We’ve all got different lenses. So, you’ve got health models, social web models, school and education model… so I think that’s where it can get complicated.” [SW1]*

*“Then siloing I reckon is probably one of the challenges… So, the way that health handles information is very different to the way that Department of Education handles information. So, getting all of those people to talk to each other and share information appropriately is hard… there are siloes everywhere.” [SW4]*

##### Theme 2. Coordination and Stability of the Workforce

Coordination of services within the SBIC model was seen to be dependent on the people who staffed them. Changes in staff was described as a critical factor to the success of implementation with the model being vulnerable when “*charismatic people”* [DNG] or ‘champions’ left to pursue other opportunities. In two schools, the principal changed. The time required to recruit to this role lent fewer opportunities for a warm handover, which led to differences in expectations from the outset. One participant reflected on a need to prepare schools for what the SBIC would involve including a shared understanding about the purpose of the clinic and what outcomes could be expected. In addition, the participant believed that professional development sessions about trauma-informed practice would be a beneficial part of this preparation.

Primary healthcare services played an integral role in SBIC models with GPs and WHIN coordinators providing stability and an important connection to the health system particularly in rural communities where access to specialists could be limited.

#### Strategy 5: Creating an Enabling Environment

An enabling environment is required for all the four previous strategies to become operational. This strategy refers to an environment that brings stakeholders together to undertake transformational change and includes changes in workforce duties, management structures, information systems, funding platforms and policy [14]. In the following section the themes related to creating and enabling environment are discussed.

##### Theme 1: Leadership

Ongoing cross-sector leadership, persistence, and excellent communication skills were considered necessary for the health and education sectors to collaborate. The characteristics described were of *“stubborn determination”* [RGP1] and *“sheer perseverance”* [DCP]. Participants spoke of a sense of *“duty to the community”* [RGP] to *“improve and protect the health and development of children”* [DCP].

Most participants felt that schools went beyond expectations in creating a welcoming environment for the different sectors to work collaboratively. Schools often provided administrative support and were key to engaging families to attend appointments.

The Aboriginal community described their vision of the importance of shared leadership for SBIC models:

*“That’s with the health department and education coming together, my original [idea] of that was, no one had ownership. It was shared. [Education] is not totally responsible for the program and neither is health; it’s a shared program.”* [AC1]

##### Theme 2. Commitment of Stakeholders and Staff

Commitment from stakeholders and staff were a cornerstone for successful SBIC implementation. This included agreement from school principals to release social workers, school counsellors or WHIN coordinators to participate in clinical work. Stakeholders cast a broad net to form cross-sector partnerships. For example, in School A an NGO was partnered with to provide social work support. At YGS, a private paediatrician, occupational therapist and speech pathologist were contracted to the SBIC.

Participants described the passionate characteristics of SBIC staff and how they went beyond their roles to achieve positive outcomes for families.

*“So I actually drive them to the service. Umm, I know it’s outside my role, but I tend to do it.”* [AEO]

“*I’ve met with [other medical staff] and they are all donating extraordinary amounts of [their] time into this work to set this system up.”* [DNG]

##### Theme 3. Operational Processes and Resourcing

Embedding processes within school policy was seen as a key operational mechanism in sustaining SBIC models. There was discussion about modernising how teams work together using data management tools or digital technology across the sectors to improve accountability and governance, “*having systems in place that are not dependent on one person remembering to collect the data*”[RES].

Some of the SBIC programs had developed MOUs to clarify roles and responsibilities as well as funding agreements. Most SBIC programs had developed written documents outlining processes. Evaluation processes were variable across sites mainly due to resource constraints. Each SBIC did however demonstrate a willingness to share data collection and evaluation systems with other sites.

### Mapping of Thematic Analysis to Looman’s Implementation Strategy

After the analysis based on the IPCHS framework, each theme was matched to Looman’s implementation strategies. An inductive process was then used to draw out strategies that would be useful when implementing SBIC models. This information is summarised in Table 3.

**Table 3:** Summary of themes mapped to IPCHS framework and Looman’s implementation strategies with implementation actions for SBIC models.


IPCHS FRAMEWORK	THEMES FROM ANALYSIS	LOOMAN’S IMPLEMENTATION STRATEGIES	SBIC IMPLEMENTATION ACTIONS

**Strategy 1: Engaging and Empowering People and Communities**	Theme 1: Community-Driven Models of Care Theme 2. Improved Access to Healthcare Theme 3. Positive outcomes for children and families Theme 4: Connection Theme 5. Marginalised Population	1. Leadership and governance: Applying collaborative governance by engaging all stakeholders	Community consultation and co-design

**Strategy 2: Strengthening governance and accounatbility**	Theme 1: Integration of Systems Theme 2. Developing a Robust Evidence Base	1. Leadership and governance: Applying collaborative governance by engaging all stakeholders	Policy and governance alignment Develop committee of leaders and community reference group

**Strategy 3: Reorienting the model of care**	Theme 1. Shifting Healthcare to Schools Theme 2: Cultural Safety	1. Workforce: Build a multidisciplinary team culture with mutual recognition of each other’s roles	Develop a ‘School Health Team’ within each school Understanding each other’s role Joint clinical meetings and professional development sessions Co-location

		2. Service delivery: Incremental growth model	Stepwise implementation with regular review process

		3. Service delivery: Balance between flexibility and formal structures of integration	Flexible approach with structure around roles and responsibilities

**Strategy 4: Coordinating services within and across sectors**	Theme 1: Integrated care Theme 2. Coordination and Stability of the Workforce	1. Workforce: Stimulate the development of new roles and competencies for integrated care	Develop new ‘integrator’ roles Develop skills for working in SBIC

**Strategy 5: Creating an enabling environment**	Theme 1: Leadership Theme 2. Commitment of stakeholders and staff Theme 3. Operational processes and resourcing	1. Leadership and governance: Distributed leadership throughout the system	Shared leadership across sectors

		2. Financing: Securing long-term funding and innovative payments	Leaders to work collaboratively to ensure sustainability

		3. Overarching mechanism: Alignment work across the different sectors	Formalising roles and responsibilities e.g. MOU Policy and process alignment

		4. Information and Communications Technology (ICT): Developed to support collaboration and communication	2a. Adopt integrated digital systems

		5. Information and Research: Feedback loops and a continuous monitoring system	3a. Resourcing for evaluation and research


## Discussion

The IPCHS framework encourages a lifespan approach to health with a shift from curative or treatment focused healthcare to health prevention, promotion and protection [14]. This framework was used to understand why and how SBIC models could improve access and engagement with health services for children experiencing physical health, developmental and behavioural concerns. Looman’s implementation strategies was used to contextualise the facilitating factors and barriers identified from the thematic analysis to ascertain strategies for scaling up the model of care within NSW. These findings have been summarised in Table 3 and will be elaborated on here.

Strategy 1 of the IPCHS framework is about engaging and empowering people and communities to make effective decisions about their own health [14]. The SBIC models described in this research were effective in improving access to healthcare due to the convenience and familiarity afforded to families by delivering services at schools while fostering a sense of connection within communities [26,27]. If we consider Looman’s implementation strategies, these findings point to the relevance of applying collaborative governance by engaging all stakeholders through community consultation and co-design [16]. The Aboriginal community were closely involved with the design of the SBIC programs, liked the collaborative nature of the model and believed that it was a more efficient way to utilise existing resources [28,29].

Strategy 2 of the IPCHS strategy relates to strengthening governance and accountability through a participatory approach in decision-making and evaluation. This research showed that working across sectors presented challenges around system harmonisation. Implementation strategies to address these challenges include prioritising extensive and continuous alignment work at a macro (system) level, meso (organisational) level and micro (clinical) level [16,30,31]. At the macro level cross-sector collaboration and alignment through leadership, policy and governance is required [32,33]. At a meso and micro level, applying collaborative governance and distributing responsibility across sectors from the outset lays the groundwork for shared responsibility and sustainability of the model [16,34]. Shared responsibility can be facilitated through two types of committees. At a meso level, a committee of leaders representing each sector can ensure that the purpose of the SBIC is clear and oversee operational processes, readiness for implementation, documentation, and navigate roadblocks [35]. At a micro level, a community reference group can oversee and guide local implementation [36,37]. It is essential that these two committees communicate with each other [37].

The success of SBIC programs in reorienting health delivery to schools, so that access to services is improved, relies on developing a strong multidisciplinary team culture (IPCHS Strategy 3: Reorienting the model of care) [37]. Participants proposed a core multidisciplinary team or a ‘School Health Team’, which included Aboriginal staff, who worked to create a ‘collaborative space’ within schools [38,39,40,41]. Over time, understanding and integration grows within teams but tensions that arise can threaten the stability of the partnership [37]. An implementation strategy to shape a positive team culture incudes joint case discussions and professional development sessions, which facilitate communication and an understanding each other’s role [16]. Co-location of professionals can additionally enhance team integration through improved frequency and quality of communication [42].

In attempting to reorientate traditional healthcare models (Strategy 3), an incremental growth model where changes occur in a stepwise fashion, leaving time for processes to be reviewed and modified, is a more successful approach to implementation [37]. Furthermore, a balance between flexibility and formal structures is required as a degree of structure is required to outline the division of tasks, and roles and responsibilities within teams [27].

Coordinating services within and across sectors is an important strategy within the IPCHS framework (Strategy 4). To coordinate the complex needs of those seen in SBIC settings, the development of new roles and competencies requires consideration. A theme that emerged was the importance of ‘integrators’, which was often an extension of existing roles [16,43]. For example, social workers and school counsellors often extended their skills to coordinate the SBIC program and provide service navigation, consistent with evidence from other such models which demonstrate that such integrators are central to the effectiveness of integrated care models [43].

Depending on a community, when scaling up, it may be that one SBIC coordinator or one SBIC team works across several schools. In this research, the skill set required of a service navigator was thought to belong to a number of professional roles [43]. As such, and in line with the concept of utilising existing resources within a community, various professionals could act as a service navigator e.g. school counsellor, WHIN coordinator, social worker, GP or community workers. Those working within SBIC could also be encouraged to seek specific training in paediatrics and youth health [44].

In the IPCHS framework, Strategy 5: creating an enabling environment, refers to the overarching factors necessary for the previous four strategies to become operational. Strong leadership and governance are important facets of this strategy [45,46]. For successful implementation of SBIC, leadership has to be distributed throughout the system [41,45]. This can be promoted through the formation of ‘leaders committees’ and ‘community reference committees’, as described for Strategy 2. In addition, sector leaders can provide governance structures and seek shared funding models to ensure sustainability through reliable funding streams [16,40,46]. Integration can be further enabled by preparing manuals and MOUs which create clarity about goals, outcomes, roles and responsibilities [40,46]. Other ways to create enabling environments includes resourcing the development of ICT and research capacity to support data sharing, collaboration, communication and evaluation [47].

Overall, this research shows that SBIC programs within NSW can improve access and engagement for with health services. The IPCHS framework and Looman’s implementation strategies were used to understand how partnerships between the health and education sectors could make significant contributions to how people experience health and care while reorienting health services based on the needs of communities [14,16]. SBIC models are valued by communities, creating impetus to scale up the initiative:

*“If you use Aboriginal culture, spread the sunshine; you spread it, you don’t keep it to yourself.”* [AC1]

### Limitations

SBIC programs within NSW have formed independently of each other and are small in number. This produces limitations in terms of the breadth of this research, which was optimised by including a COP in the study.

## Conclusions

Strategies for implementing SBIC models across NSW have been identified and include community consultation and co-design to create a program that is effective, culturally-safe and durable in engaging children and their families and providing improved access to healthcare. Multidisciplinary team culture needs to be actively and continuously built through avenues such as meetings and shared professional development sessions. The skill set and make-up of the multidisciplinary requires consideration, particularly as new competencies and roles may be required e.g. linkers and coordinators. Collaborative and shared leadership across sectors is particularly important in providing guidance and direction while securing stable funding streams. Overarching mechanisms for SBIC implementation include alignment of operational systems through protocols and policies while maintaining a balance between structure and flexibility.

## Appendix A: Interview Guide for Phase 2

Generally, the primary questions analysed were, with slight variation, to all participants.

1. *What has been your involvement in Yudi Gunyi School (YGS) or Ngaramadhi Space (NS)?*
2. *What is your understanding of the YGS/NS model?*
3. *Can you describe how the multi-disciplinary (education & health) team works together?*
4. *What are the benefits and/or challenges of the YGS/NS model?*
5. *What changes or developments have led to the current model?*
6. *What has been the experience of the students and families of YGS/NS?*
7. *What are the critical or key elements of the YGS/NS model?*
8. *How do you think these critical elements could be adopted or adapted for use in other school settings?*

## Appendix B: Interview Guide for Phase 3

Generally, the primary questions analysed were, with slight variation, to all participants.

1. *What has been your involvement in the school clinic?*
2. *Can you chart a timeline of how the school clinic was established?*
3. *What facilitated the establishment of the clinic?*
4. *What barriers did you come across when establishing the clinic?*
5. *How were barriers overcome?*
6. *What were your expectations when setting up a school clinic?*
7. *Have these expectations been met or not met? Why do you think this is the case?*
8. *Who accesses the clinic at the moment? Could anyone else access the clinic?*
9. *What have been the benefits of forming a multidisciplinary clinic?*
10. *What could be done better?*
11. *Overall, do you think that the school clinics are worthwhile?*
12. *Do you think the school clinics should be replicated in other settings?*
13. *What would be needed to replicate the model?*
